# Microfluidic-engineered glucose-responsive microparticle depot for smart insulin delivery

**DOI:** 10.1080/10717544.2026.2726633

**Published:** 2026-09-03

**Authors:** Zhihan Li, YanWen Zhang, Chuanpin Chen

**Affiliations:** a Department of Pharmacy, Xiangya School of Pharmaceutical Sciences, Central South University, Changsha, China

**Keywords:** Glucose-responsive, insulin delivery, microsphere, microfluidics, type 1 diabetes mellitus

## Abstract

Diabetes mellitus, chronic metabolic disease marked by persistent hyperglycemia resulting from inadequate insulin production or impaired action. Despite advancements in glucose-responsive insulin delivery systems, challenges persist such as limited responsiveness to dynamic glucose fluctuations, in vivo biocompatibility, and production scalability with uniform performance. However, insulin-loaded microspheres combined with microfluidics-based technology improve the subcutaneous insulin reservoir and homogeneous microsphere production rate. This study presents a microfluidic-engineered glucose-responsive controlled-insulin release microsphere using Glucose oxidase (GOx) as the glucose-sensing unit and a hybrid carrier composed of poly (lactic-co-glycolic acid) (PLGA) and acid-sensitive acetalated dextran (Ac-dex). The microfluidic-based fabrication method ensured uniform and controllable microsphere size and stable performance with satisfactory batch-to-batch reproducibility. In Streptozotocin (STZ)-induced diabetic mice, the microspheres maintained normal blood glucose levels for at least 20 hours. Absence of additional treatment-induced organ toxicity, systemic inflammation, and low hypoglycemic index ensured that the formulation is safe. Besides, this study provides a promising strategy for closed-loop insulin therapy, with potential clinical applications in improving glycemic control and reducing the burden of diabetes management.

## 1. Introduction

Diabetes mellitus is a chronic metabolic disorder characterized by sustained hyperglycemia resulting from defective insulin secretion, impaired insulin action, or both (Harreiter and Roden 2023; Diagnosis and Classification of Diabetes: Standards of Care in Diabetes-2024 2024). The global prevalence of diabetes has been increasing annually, currently affecting approximately 589 million individuals worldwide, and is projected to rise to 783 million by 2045 (Saeedi and Petersohn, 2019). For type 1 diabetes (T1D) and advanced type 2 diabetes (T2D), subcutaneous insulin injection remains the standard therapeutic approach (Akil et al. 2021). However, this conventional glucose-regulation method presents several limitations: frequent administration requirements leading to poor patient compliance, significant glycemic fluctuations, and substantial risks of hypoglycemia that may trigger severe complications including shock and mortality (McCall and Farhy 2013).

To overcome these limitations, substantial research efforts have been directed toward developing advanced insulin delivery strategies, encompassing both molecular engineering of insulin analogs for prolonged action (Hemmingsen et al. 2021; Rosenstock et al. 2024) and material-based approaches for sustained release (Shen et al. 2020; Yao et al. 2022). Notably, glucose-responsive delivery systems (Shen et al. 2020; Wang et al. 2020; Martínez-Navarrete et al. 2024) have emerged as a particularly promising solution, as they can recapitulate the physiological glucose-sensing mechanism of pancreatic *β*-cells to achieve real-time, closed-loop insulin release that is precisely coupled to blood glucose levels. Numerous glucose-responsive insulin delivery platforms have been developed, primarily utilizing glucose-sensing elements such as GOx (Volpatti et al. 2020; Chai et al. 2020; Zhou et al. 2021; Zhang et al. 2022, 2020), phenylboronic acid (PBA) (Yang et al. 2022; Wang et al. 2023; Xian et al. 2024; Ji et al. 2024), and Concanavalin A (ConA) (Xu et al. 2022; Bai et al. 2022; Bercea and Lupu 2024). Among these, GOx has been extensively employed due to its excellent biocompatibility, high specificity, and minimal requirement for complex modification. When blood glucose levels rise, GOx catalyzes the conversion of glucose to gluconic acid, subsequently lowering the local pH. This pH reduction can be coupled with acid-sensitive carriers (Rizwan et al. 2017; Yao et al. 2022) to achieve on-demand insulin release.

Nevertheless, several challenges remain to be solved, including carrier biocompatibility, capability for multiple responsive releases, scalability for mass production, and formulation stability. Conventional nanoparticle-based systems (Volpatti et al. 2020; Shen et al. 2022; Liu et al. 2023) often suffer from single-use limitations and poor sustained release. Meanwhile, to improve insulin loading efficiency, nanoparticle carriers are frequently fabricated using positively charged materials, which inevitably introduces toxicity concerns (Wang et al. 2019; Liu et al. 2023; Zhang et al. 2024). On the other hand, microneedle patches (GhavamiNejad et al. 2019; Chen et al. 2019; Makvandi et al. 2021; Yang et al. 2022) face challenges such as protein denaturation during fabrication, stringent storage requirements, and high inter-subject variability due to skin barrier differences. Moreover, both systems encounter scalability hurdles in mass production.

**Figure 1.**
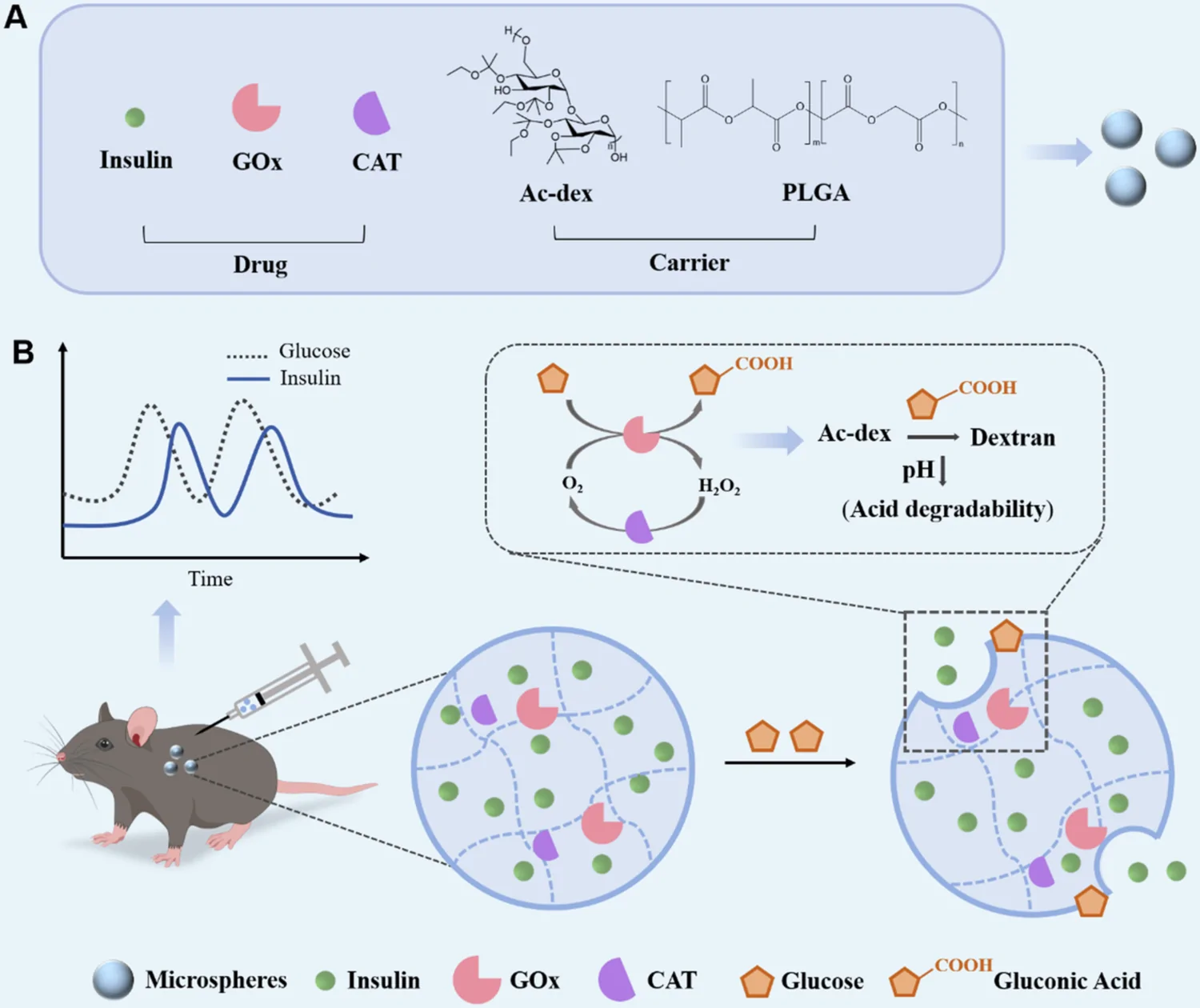
Schematic representation of the Glucose-responsive microspheres. (A) Drug and carrier composition of glucose-responsive microspheres. (B) Mechanism of insulin release in response to blood glucose levels. PLGA forms the microsphere scaffold. When blood glucose rises, GOx converts glucose to gluconic acid, lowering the local pH and triggering degradation of Ac-dex. This results in outer-layer insulin release. If blood glucose levels continue to rise, Ac-Dex will undergo further degradation, thereby sustaining insulin release. Conversely, when blood glucose returns to normal levels, insulin release ceases, achieving glucose-responsive drug delivery.

Here, we developed an insulin-loaded microsphere system using microfluidic technology, employing PLGA (Su et al. 2021; Muddineti and Omri 2022) and Ac-dex (Bachelder et al. 2017; Wang et al. 2021) as carriers with GOx as the glucose-responsive component. In this design, PLGA serves as the matrix material while Ac-dex functions as the pH-sensitive carrier. As illustrated in Figure 1, elevated glucose levels trigger GOx-mediated gluconic acid production, leading to gradual pH-dependent degradation of Ac-dex from the exterior to the interior, thereby releasing encapsulated insulin. These microspheres can serve as subcutaneous insulin depots, achieving glucose-regulated insulin release and maintaining normoglycemia for over 20 hours in STZ-induced diabetic mice (Singh and Gholipourmalekabadi, 2024). All selected materials exhibit excellent biocompatibility, with catalase (CAT) incorporated to neutralize H_2_O_2_ generated during GOx catalysis. The microfluidic fabrication approach ensures uniform microsphere size and performance characteristics while enabling large-scale production (Su et al. 2021; Wang et al. 2023).

This glucose-responsive insulin delivery system employing dual carriers exhibits several remarkable advantages: (1) The microfluidic-engineered drug-loaded microspheres demonstrate monodisperse characteristics and excellent scalability for industrial production; (2) The synergistic combination of PLGA and Ac-dex carriers not only ensures biocompatibility but also provides pH-triggered release properties, which when coupled with the GOx-CAT enzymatic reaction system, achieves dynamic glucose-dependent insulin release kinetics; (3) Most significantly, animal studies have demonstrated prolonged maintenance of euglycemia (>20 hours) while simultaneously addressing the critical limitations of conventional approaches—specifically, the cytotoxicity of cationic nanocarriers and the stability challenges of protein drugs in microneedle systems. This innovative platform, integrating real-time glucose monitoring, stimulus-responsive release, and sustained glycemic regulation, represents a conceptual advance toward the development of closed-loop insulin delivery systems.

## 2. Materials and Methods

### 2.1. Materials

The Insulin (≥25 IU/mg) and STZ were purchased from Beijing Solarbio Science & Technology Co., Ltd. (Beijing, China). GOx and CAT were purchased from Shanghai Yuanye Bio-Technology Co., Ltd (Shanghai, China). Dextran (Mw 20000), DMSO (Extra Dry), 2-ethoxypropene, pyridinium *p*-toluenesulfonate (PPTS), and triethylamine were purchased from Energy Chemical, Senrise Technologies Co., Ltd. (Anhui, China). Poly (lactic-co-glycolic acid) (PLGA, lactide: glycolide = 50: 50), -COOH capped, Mw 40000, was purchased from Jinan Daigang Biomaterial Co., Ltd. (Shandong, China), Cy5-insulin was purchased from Qiyue Biotechnology Co., Ltd. (Xian, China).

### 2.2. Synthesis of Ac-dex

Dextran (1.0 g) and PPTS (15.6 mg) were charged into a flame-dried flask under a nitrogen atmosphere. DMSO (Extra Dry, 10 mL) was added, and the mixture was stirred for 5 minutes to ensure complete dissolution of dextran. Subsequently, 2-ethoxypropene (4.16 mL) was introduced, and the reaction was allowed to proceed under stirring at room temperature for 1 hour. The reaction was quenched by the addition of triethylamine (1 mL). The resulting solution was then slowly added dropwise into ice-cold deionized water (100 mL) containing 0.1% (v/v) triethylamine. The mixture was centrifuged at 6000 rpm for 10 minutes to isolate the product. The white precipitate was collected, washed twice with deionized water (0.1% triethylamine), and lyophilized to afford the final protected dextran derivative as a white powder.

### 2.3. Characterization of Dextran and Ac-dex by ^1^H NMR and FTIR Spectroscopy

The Dextran was dissolved in D_2_O at a concentration of 10 mg/mL. The Ac-dex sample was dissolved in deuterated dimethyl sulfoxide (DMSO-d₆) at a concentration of 10 mg/mL. The mixture was vortexed for 30 seconds and sonicated for 10 minutes to ensure complete dissolution. The solutions were loaded in NMR tubes and tested using an NMR spectrometer (AVANCE III 500 MHz, Bruker Scientific Technology Co., Ltd. Swiss.). FTIR spectra were recorded using the KBr pellet method on a Thermo Scientific Nicolet iS5 spectrometer (Thermo Fisher Scientific, USA) over the wavenumber range of 4000–400 cm^−1^ with a resolution of 4 cm^−1^ and 32 scans. All measurements were performed at room temperature.

### 2.4. Fabrication of two-phase flow-focusing microfluidic chips

The two-phase flow-focusing microfluidic chip was fabricated using standard soft lithography techniques. First, the microchannel pattern was designed using Adobe Illustrator, with individual phase channels set to a width of 100 μm and the outlet channel (exit) expanded to 260 μm. The design was then printed onto a photomask for subsequent lithography.

**Figure 2.**
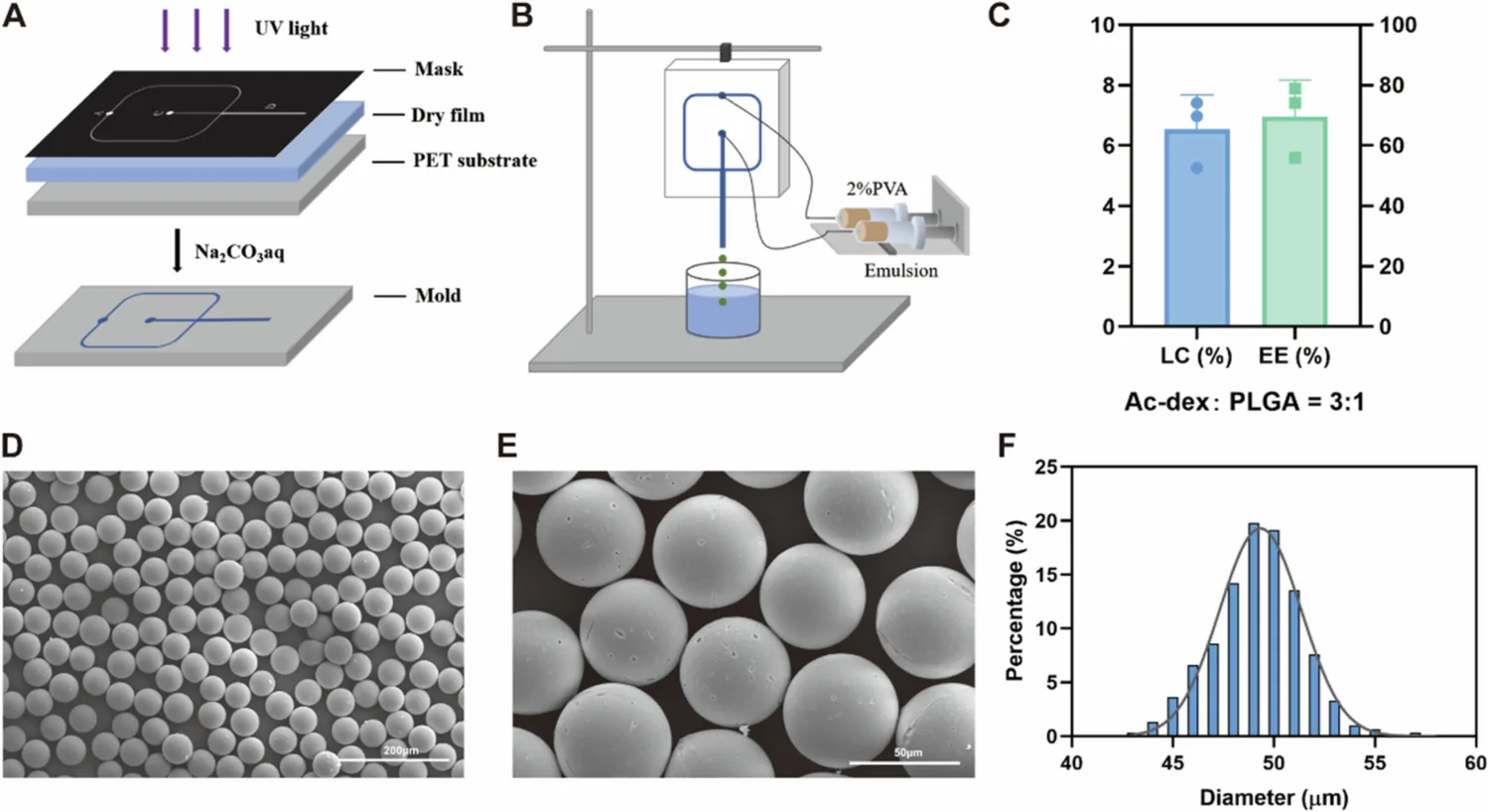
Preparation and characterization of microspheres. (A) Fabrication process of the microfluidic chip mold. (B) Schematic diagram of biphasic microfluidic device for microsphere preparation. (C) The LC and EE of Microspheres of Ac-dex: PLGA= 3: 1. Data are presented as mean ± s.d. (*n* = 3). (D) The SEM image of microspheres (scale bar, 200 µm). (E) SEM image of microspheres (scale bar, 50 µm). (F) Histogram of the frequency distribution of the diameter of microspheres and Gaussian analysis.

A 25-μm-thick Dupont dry film photoresist (Riston FX900, DuPont Co., Ltd., Wilmington, DE) was laminated onto a polyethylene terephthalate (PET) substrate (0.175 mm, Kaivo Optoelectronic Technology Co., Ltd., Zhuhai, China). The photoresist-coated PET was exposed to UV light (HT-3040, Shijiazhuang Ourpcb Co., Ltd., Shijiazhuang, China) through the photomask for 180 seconds. The uncured regions were then dissolved in a 0.85% (w/v) Na₂CO₃ solution, yielding the master mold for the microfluidic chip (Figure 2A).

Polydimethylsiloxane (PDMS) prepolymer and curing agent (10:1 ratio) were thoroughly mixed, degassed, poured onto the mold and thermally cured at 60 °C for 2 hours. The cured PDMS was then removed from the mold, cut to the desired dimensions and shape, and punched using a 0.70 mm biopsy core (Anhui, China) for the inlet and outlet parts. The structured PDMS layer and a flat PDMS slab were activated via corona treatment and permanently bonded to form the sealed microfluidic device. Finally, polytetrafluoroethylene (PTFE) tubing was connected to the inlets for fluidic interfacing with a syringe pump (Figure 2A).

### 2.5. Preparation of insulin-loaded microspheres

Insulin (10 mg/mL), GOx (12.5 mg/mL) and CAT (2.5 mg/mL) were dissolved in 0.1 mol/L hydrochloric acid solution as a water-phase solution. Ac-dex (30 mg/mL) and PLGA (10 mg/ml) were dissolved in dichloromethane as oil phase solutions. The volume ratio of the water:oil phase solution was 1:6. After mixing, use an ultrasonic homogenizer (JY96-IIN, Ningbo Scientz Biotechnology Co., Ltd., Ningbo, China) to perform ultrasonic treatment with 60% power. Under the condition of ice bath, ultrasonic treatment was performed for 1 second, with an interval of 1 second, for a total of 1 minute, to obtain a yellowish turbid emulsion. Using this emulsion as internal phase and 2% PVA solution as external phase, the sample was injected into the chip by an injection pump, and the flow rate ratio of internal and external phases was 3:5, and the flow rate was adjusted according to the actual situation until stable and uniform droplets were collected. The receiving liquid was a mixture of 2% PVA and 5% NaCl. The droplets were stirred while receiving, solidified for 4 hours, and completely formed into microspheres, which are washed with pure water and collected (Figure 2B).

### 2.6. SEM image of insulin-loaded microsphere

The prepared microspheres were washed thoroughly with deionized water and collected by centrifugation at 1000 × g for 5 minutes. Subsequently, the samples were pre-frozen at −20 °C and then transferred to a vacuum freeze drier (LC-10N-80A, Shanghai Lichen Instrument Technology Co., Ltd., Shanghai, China) for drying. After drying, the microspheres were mounted onto the sample stage using conductive adhesive. The sample stage was then placed into an ion sputtering coater to deposit a 5–10 nm gold layer. Finally, the sample stage was inserted into a SEM. Initial observation was carried out at low magnification to locate appropriate fields of view, followed by a stepwise increase in magnification for detailed examination. Imaging parameters, including acceleration voltage and working distance, were adjusted to obtain clear images.

### 2.7. The loading capacity (LC) and encapsulation efficiency (EE) of insulin-loaded microspheres

The insulin content was determined using the Bradford protein assay with the following procedure: lyophilized microspheres were dissolved in dichloromethane and sonicated for 10 minutes to ensure complete dispersion. Subsequently, 0.1 M hydrochloric acid was added, followed by vortex mixing for 5 minutes and centrifugation at 4000 × g for 10 minutes at 4 °C. The resulting supernatant was transferred to a 96-well plate and mixed with Coomassie Brilliant Blue reagent for 5 minutes. Absorbance was measured at 595 nm using a microplate reader (infinite M200 PRO, Austria). A standard curve was generated using purified insulin (0.1–100 μg/mL) for quantitative analysis. The LC and EE were calculated as follows:

(1) $$\mathrm{DL}(\%)=\frac{\mathrm{Mass of encapsulated insulin}}{\mathrm{Total mass of microspheres}}\times 100$$

(2) $$\mathrm{EE}(\%)=\frac{\mathrm{Mass of encapsulated insulin}}{\mathrm{Mass of insulin initially added}}\times 100$$

### 2.8. Circular dichroism (CD) characterization of Loaded insulin

The secondary structure of insulin was analyzed using CD spectroscopy. Insulin-loaded microspheres were dissolved in dichloromethane and extracted with 0.01 M acetic acid solution through vigorous vortexing for 5 minutes. After phase separation by centrifugation (4000 × g, 10 minutes, 25 °C), the aqueous phase containing insulin was collected. The insulin concentration in the supernatant was quantified using the Bradford protein assay. For CD measurements, an insulin reference solution was prepared at identical concentration in 10 mM phosphate buffer (pH 7.4). CD spectra were recorded from 190 to 260 nm using a 1 mm pathlength quartz cuvette at 25 °C, with data representing the average of three consecutive scans.

### 2.9. In vitro insulin release studies for insulin-loaded microspheres

Four aliquots of microspheres (10 mg each, *n* = 3) were incubated in 1 mL PBS containing different glucose concentrations (0, 100, 200, and 400 mg/dL) at 37 °C. At predetermined time intervals (0, 4, 6, and 8 hours), 50 μL aliquots of supernatant were collected and the insulin content was quantified using the Bradford assay. Additional microsphere samples (10 mg, *n* = 3) were suspended in PBS (pH 7.4, 1 mL). The glucose concentration was sequentially increased from 100 to 200 and finally 400 mg/dL at 1 hour intervals. Insulin release was monitored following the same analytical protocol described above. Then, the microspheres were alternately soaked in 100,400 mg/dl glucose solution, and the changes of insulin release were measured.

### 2.10. Enzyme stability and leakage studies

Microspheres (approximately 20 mg per sample) were dispersed in 1 mL of phosphate-buffered saline (PBS, pH 7.4) and incubated at 37 °C under gentle shaking (100 rpm). At predetermined time intervals (0, 1, 3, 5, 7, and 14 days), aliquots of the suspension were collected and centrifuged to separate the microspheres from the supernatant. The total protein concentration in the supernatant was determined using the Bradford assay, with bovine serum albumin (BSA) as the standard, and the absorbance was measured at 595 nm using a microplate reader (Synergy H1, Agilent Biotek, USA). The amount of enzyme leaked from the microspheres was expressed as the percentage of total loaded protein detected in the supernatant.

To measure the residual activities of GOx and CAT retained within the microspheres, the microspheres were first dissolved in DCM, and the enzymes were extracted into PBS. The resulting aqueous phase was collected for subsequent activity assays. GOx activity was measured using the Amplex Red Glucose Oxidase Activity Assay Kit (Beyotime, P2747S). Briefly, 20 µL of the extracted sample or GOx standard was mixed with 80 µL of Amplex Red reaction working solution in a 96‑well plate. The mixture was incubated at 37 °C in the dark for 20 minutes. The absorbance was then measured at 570 nm using the Synergy H1 microplate reader. The GOx activity was calculated based on the standard curve. CAT activity was measured using the Catalase Assay Kit (Beyotime, S0051) according to the manufacturer's protocol. The assay is based on the reaction of catalase with H₂O₂; the remaining H₂O₂ is detected by a chromogenic probe. The extracted sample was incubated with the provided reagents at 25 °C for 20 minutes, and the absorbance was measured at 520 nm using the Synergy H1 microplate reader. One unit of CAT activity is defined as the amount of enzyme that catalyzes the decomposition of 1 µmol of H₂O₂ per minute at 25 °C. The residual enzyme activity was calculated as the percentage of activity at each time point relative to the initial activity (day 0).

### 2.11. In vitro degradation of PLGA/Ac‑dex hybrid microspheres

Microspheres (ca. 20 mg each) were incubated in glucose solutions at concentrations of 0, 100, 200, and 400 mg/dL, respectively, in sealed glass vials placed in a temperature‑controlled orbital shaker at 37 °C with a constant agitation speed of 100 rpm. At predetermined time intervals (0, 2, 4, 6, and 8 hours), the vials were retrieved, and the microspheres were collected by centrifugation at 1000 × g for 10 minutes. The supernatant was discarded, and the microspheres were washed twice with deionized water to remove any residual glucose and salts, followed by lyophilization under vacuum at −20 °C for 24 hours to constant dry weight. The dried microspheres were dissolved in 1.0 mL of HPLC‑grade tetrahydrofuran (THF) to obtain a final concentration of approximately 5–10 mg/mL. Prior to GPC analysis, all sample solutions were filtered through 0.45 μm syringe filters. The molecular weights of the degraded samples were determined by gel permeation chromatography (GPC; Agilent 1260 system equipped with a refractive index detector, PLgel columns, THF as the mobile phase at 1.0 mL/min, 35 °C), calibrated with polystyrene standards.

### 2.12. Hemolysis assay

The hemolytic activity of MPs was evaluated under physiological (pH 7.4) and acidic (pH 6.5) conditions to simulate the normal microenvironment and the hyperglycemia-associated microenvironment, respectively. Sterile anticoagulated sheep blood was centrifuged at 1000 × g for 10 min at 4 °C to isolate red blood cells (RBCs). The erythrocyte pellet was washed three times with sterile phosphate-buffered saline (PBS, pH 7.4), and the final pellet was resuspended in PBS to prepare a 2% (v/v) erythrocyte suspension. Microspheres were dissolved in PBS at concentrations of 1.5 and 10 mg/mL, and the solutions were prepared at pH 7.4 and pH 6.5, respectively. PBS at pH 7.4 and pH 6.5 served as negative controls, whilst deionized water served as the positive control. For each test, 100 µL of the RBC suspension was mixed with 900 µL of the sample or control solution in a 1.5-mL microcentrifuge tube. The mixtures were incubated at 37 °C for 2 hours in a shaking water bath at 100 rpm. After incubation, the tubes were centrifuged at 1000 × g for 10 minutes to sediment intact RBCs. Subsequently, 200 µL of each supernatant was transferred to a 96-well flat-bottom plate, and the absorbance was measured at 540 nm using a microplate reader (Synergy H1, Agilent BioTek, USA). The hemolysis percentage was calculated according to the following equation:

(3) $$\mathrm{Hemolysis}(\%)=\frac{A\;\mathrm{sample}-A\;\mathrm{negative}}{A\;\mathrm{positive}-A\;\mathrm{negative}}\times 100$$

### 2.13. In vivo antidiabetic studies for Glucose-responsive microspheres in T1D mice

Male C57BL/6 mice (6–8 weeks old, purchased from Hunan SJA Laboratory Animal Co., Ltd. (Laboratory Animal Production License Number: SCXK (Xiang) 2021-0002)) were acclimatized to the housing conditions for 2–3 days prior to experiments. All animal experiments were conducted in accordance with the ARRIVE guidelines (Percie du Sert et al. 2020) and were approved by the Animal Ethics Committee of the Research Institute of Quality Inspection of Products in Hunan Province, China. Following a 12-hour fasting period (with free access to water), diabetes was induced by intraperitoneal injection of STZ (150 mg/kg). After 2 hours, standard chow and glucose-supplemented drinking water (15 g/L) were provided. Three days post-injection, non-fasting blood glucose levels were measured via tail vein sampling using a glucometer. Blood sampling was performed on mice under light isoflurane anesthesia (induced at 3% and maintained at 1.5%–2% in oxygen). Mice with random blood glucose levels exceeding 300 mg/dL were considered T1D. Throughout the study period, all animals had ad libitum access to food and water, and the experiments were started in the morning.

### 2.14. Blood glucose control study in T1D mice

Insulin-loaded microspheres with a drug loading content of 6.9% ± 0.5% were used in this study. All microsphere doses are expressed as insulin-equivalent doses to enable direct comparison with free insulin controls. T1D mice were randomly divided into experimental groups and administered subcutaneous injections of: (1) PBS (pH 7.4) as vehicle control, (2) free insulin (10 mg/kg), or (3) insulin-loaded microspheres (20 mg/kg insulin-equivalent dose). All animals- maintained ad libitum access to food and water throughout the study period. Blood glucose levels were monitored at predetermined time intervals using a commercial glucometer (Yuwell 580). Measurements were obtained via tail vein puncture. Blood sampling was performed on mice under light isoflurane anesthesia.

### 2.15. Determination of plasma insulin levels in T1D mice after administration

T1D mice were randomly divided into two experimental groups and received a single subcutaneous injection of either free insulin (10 mg/kg) or insulin-loaded microspheres (20 mg/kg insulin-equivalent dose). Blood samples were collected via the medial canthus at pre-determined time points: 0 (pre-injection), 0.5, 1, 2, 4, 8, 12, and 24 hours post-injection. Blood was collected into heparinized microcentrifuge tubes and immediately centrifuged at 1000 × g for 15 minutes at 4 °C to separate the plasma. The supernatant was carefully aliquoted and stored at −80 °C until further analysis. The concentration of mouse insulin in serum was measured using a Mouse Insulin ELISA Research Kit (Shanghai Kexing Commerce & Trade Co., Ltd.). The assay was performed as follows: 100 µL of standards and diluted (1:10) mouse serum samples were added to a pre-coated 96-well plate and incubated at 37 °C for 90 minutes. After washing, a biotinylated detection antibody was added and incubated for 60 minutes, followed by another wash. Streptavidin-HRP was then added and incubated for 30 minutes. After thorough washing, TMB substrate solution was added and incubated in the dark for 10–15 minutes, after which the reaction was stopped. Absorbance was immediately measured at 450 nm using a microplate reader.

### 2.16. Intraperitoneal glucose tolerance test (IPGTT) in T1D mice

The therapeutic efficacy of insulin and microspheres was evaluated in T1D mice using identical doses to those described previously. Blood glucose levels were monitored continuously until normoglycemia was restored (<200 mg/dL). Subsequently, mice received an intraperitoneal glucose challenge (1.5 g/kg), with continued monitoring of glycemic response. Age-matched healthy mice served as controls and underwent identical glucose administration and monitoring procedures simultaneously with the treatment groups. This parallel testing design enabled direct comparison of glucose homeostasis recovery between diabetic and normoglycemic animals.

### 2.17. Plasma insulin level measurement during IPGTT in T1D mice

T1D mice were administered microspheres at the previously described dosage, with blood glucose levels monitored until restoration of normoglycemia (~100 mg/dL). Subsequently, an IPGTT was performed by administering 3 g/kg glucose solution. Blood samples (40 µL) were collected via the medial canthus at baseline (0 minute) and 15, 30, 45, 60, and 90 minutes post-injection for glycemic analysis. For insulin measurement, whole blood samples were allowed to clot for 1 hour at room temperature, followed by centrifugation at 1000 × g for 10 minutes to obtain plasma. Plasma insulin concentrations were determined using the same ELISA method as described in Section 2.16, with a Mouse Insulin ELISA Research Kit (Shanghai Kexing Commerce & Trade Co., Ltd.).

### 2.18. Hypoglycemic index of normal mice

Healthy mice were subcutaneously administered either free insulin (10 mg/kg) or insulin-loaded microspheres (20 mg/kg insulin-equivalent dose) at doses identical to those used in diabetic models. Blood glucose levels were monitored continuously, and the general condition of the animals was observed for signs of hypoglycemia. The hypoglycemic index (HGI) was calculated as follows:

(4) $$\mathrm{HGI}=\frac{\Delta \mathrm{Blood Glucose}\;(\mathrm{Initial}-\mathrm{Nadir})}{\mathrm{Time to Nadir}\;(\min)}$$

### 2.19. In vivo biodistribution of labeled insulin-loaded microspheres in T1D mice

Microspheres were prepared using Cy5-conjugated insulin (Cy5-insulin) following the established protocol. After dorsal hair removal, diabetic mice were randomly divided into treatment groups receiving subcutaneous injections of either: Free Cy5-insulin, or Cy5-insulin-loaded microspheres, with doses equivalent to those described in previous experiments. In vivo fluorescence imaging was performed at predetermined time intervals (0, 2, 4, 8, 12, and 24 hours post-injection) using a multi-model animal in vivo imaging system (Aniview, Guangzhou Biolight Biotechnology Co., Ltd., Guangzhou, China). Quantitative analysis of fluorescence intensity was conducted using manufacturer-provided software, with regions of interest (ROIs) standardized across all time points and animals. Background fluorescence was subtracted from all measurements using pre-injection images as baseline controls.

### 2.20. H&E test for skin and organ tissue

Following dorsal hair removal, diabetic mice were randomly assigned to receive subcutaneous injections of: (1) PBS (pH 7.4) as vehicle control, (2) insulin, or (3) microspheres, using identical dosages to previous experiments. Treatments were administered every 24 hours for one month. On day 30 post-initial injection, mice were euthanized via cervical dislocation under deep isoflurane anesthesia (5%). Injection site skin tissue and major organs (heart, liver, spleen, lungs, and kidneys) were immediately harvested and fixed in 4% paraformaldehyde for 24 hours at 4 °C. Fixed tissues were processed through standard paraffin embedding protocols, sectioned at 5 μm thickness, and stained with H&E for histological evaluation of inflammatory responses. Additional skin sections from the injection sites were subjected to Masson's trichrome staining to quantitatively assess collagen deposition and fibrosis development using standardized image analysis protocols.

### 2.21. In vivo biosafety analysis

Whole blood samples were collected from these experimental cohorts: healthy control mice, untreated diabetic mice, and diabetic mice receiving one-month therapy with PBS, insulin, or microspheres. Complete blood counts were immediately performed using an automated hematology analyzer (Mindray BC-5100, China). For biochemical analysis, serum was separated by centrifugation at 1000 × g for 15 minutes at 4 °C and stored at −80 °C until analysis. Hepatic and renal function parameters including alanine aminotransferase (ALT), aspartate aminotransferase (AST), and alkaline phosphatase (ALP) were quantified on an automatic biochemical analyzer (Mindray BS-360E, China).

### 2.22. Statistical analysis

Data were expressed as the mean ± s.d. The statistical significance of the difference between the two groups was evaluated using a two-tailed t-test. Student's t-test, and the difference between three or more groups was evaluated using a one-way analysis of variance (ANOVA). The statistical significance was considered when *P < 0.05, **P < 0.01, ***P < 0.001 and ****P < 0.0001.

## 3. Ethical approval statement

This study was conducted in strict accordance with the ARRIVE guidelines 2.0. All experimental protocols involving animals were reviewed and approved by the Animal Ethics Committee of the Research Institute of Quality Inspection of Products in Hunan Province, China (Approval No.: JLGS-03320-2025-2-2). This research was conducted through a collaborative project between the Xiangya School of Pharmaceutical Sciences, Central South University and the Research Institute of Quality Inspection of Products in Hunan Province. Since all animal housing, experimental procedures, and related operations were physically conducted at and managed by the Research Institute's animal facility, ethical approval was properly sought and obtained from their institutional Animal Ethics Committee. This arrangement ensures direct, site-specific oversight and compliance with animal welfare standards at the location where the procedures were performed.

The approval was granted on [2025-02-20], and all in vivo experiments described in the Methods section were carried out between [2025-03-01] and [2025-04-16] at the Research Institute's facility. Additional ethical approval for supplementary experiments was obtained from the Animal Ethics Committee of the Xiangya School of Pharmaceutical Sciences, Central South University (Approval No.: 202629) on [2026-06-08], and the experiments were performed between [2026-06-10] and [2026-07-17].

All animals were housed in a specific pathogen-free (SPF) facility under controlled environmental conditions: temperature 22 ± 2 °C, humidity 50% ± 10%, and a 12-hour light/dark cycle. Mice were housed in individually ventilated cages, with a maximum of 5 mice per cage, and provided with standard laboratory chow and autoclaved water ad libitum. To minimize suffering, invasive procedures such as blood sampling were performed under general inhalation anesthesia using isoflurane (induction at 3%, maintenance at 1.5%–2%). Humane endpoints were strictly observed. At the conclusion of the study, euthanasia was performed under deep anesthesia (5% isoflurane) followed by cervical dislocation, a method confirmed to cause rapid loss of consciousness.

## 4. Results

### 4.1. Preparation and characterization of the glucose-responsive insulin microspheres

A microfluidic-engineered glucose-responsive controlled-insulin release microsphere was fabricated by using GOx and a hybrid carrier made of PLGA and acid-sensitive Ac-dex for efficient treatment of T1D. The pH-sensitive Ac‑dex was synthesized via a one-step reaction under anhydrous and oxygen-free conditions in a flame‑dried flask, affording the product in 81.3% ± 1.7% yield (Fig. S1) (Kauffman et al. 2012). To confirm the successful synthesis of Ac‑dex, the product and its precursor (dextran) were characterized by ^1^H NMR and FTIR spectroscopy. The ^1^H NMR spectrum of Ac‑dex (Fig. S2) displayed a new signal at *δ* 2.0–2.1 ppm, corresponding to the methyl protons of the acetyl groups (–COCH₃), thereby confirming the successful acetylation of the hydroxyl groups in dextran. This finding was further supported by FTIR analysis (Fig. S3). Compared with native dextran, the FTIR spectrum of Ac‑dex exhibited a markedly reduced O–H stretching band at approximately 3300 cm^−1^ and an enhanced C–CH₃ bending band at around 1370 cm^−1^. Collectively, the ^1^H NMR and FTIR results unequivocally demonstrate the successful synthesis of Ac‑dex.

The microspheres were fabricated using a microfluidic system to ensure uniform morphology, stability, and reduced burst release. The microfluidic chip mold was prepared via soft lithography (Figure 2A). After fabricating the two-phase mold, the chip was constructed using PDMS and assembled into a microfluidic device. Subsequently, the emulsion obtained by ultrasonication of the drug and carrier was employed as the internal phase, while 2% PVA solution served as the external phase, to fabricate microspheres under optimized flow rate conditions. (Figure 2B). The SEM image of microspheres prepared with Ac-dex: PLGA = 3:1 (Figure 2B and E) exhibited an intact morphology and consistent size. Figure 1F shows the percent frequency distribution of the diameter of the microsphere samples with a mean diameter and the average microparticle size was 49.2 ± 2.2 μm. The figure indicates that the microspheres show a uniform particle size distribution which is in accordance with the Gaussian distribution pattern. Quantification of insulin concentration using the Bradford test yielded 6.9% ± 0.5% LC and 73.8% ± 5.3% EE (Figure 2C), corresponding to relative standard deviation (RSD) values of 7.2%.

### 4.2. Glucose-responsive insulin-loaded microspheres formulation optimization

To identify the optimal microsphere formulation with enhanced glucose responsiveness, we systematically varied the mass ratio of Ac-dex to PLGA. Microspheres with Ac-dex: PLGA ratios of 1:1, 3:1, 5:1, and 7:1 were initially fabricated, followed by characterization of their LC and EE (Fig. S4). The LC and EE exhibited a progressive increase with higher Ac-dex content, achieving a maximum EE of 93.6%. Subsequent evaluation of insulin release profiles under varying glucose concentrations revealed distinct performance differences among the formulations (Fig. S5). The 1:1 ratio formulation demonstrated minimal insulin release within hours and lacked glucose-responsive characteristics. Conversely, the 7:1 ratio group exhibited rapid insulin release across all glucose concentrations without proper responsiveness. Notably, microspheres with intermediate ratios (3:1 and 5:1) displayed optimal glucose-dependent insulin release behavior. To assess long-term stability under physiological conditions, selected formulations (3:1 and 5:1) were incubated in PBS (pH 7.4) at 37 °C for two weeks, simulating in vivo storage conditions. As illustrated in Fig. S6, the 5:1 formulation demonstrated significant insulin leakage in neutral PBS, with cumulative release reaching 133.1 ± 5.0 µg/mL after two weeks. In contrast, the 3:1 ratio exhibited superior retention capacity, maintaining a substantially lower free insulin concentration of only 16.8 ± 2.0 µg/mL over the same period. Based on these findings, the Ac-dex: PLGA 3:1 formulation was selected for all subsequent experiments due to its optimal balance between glucose responsiveness and storage stability.

### 4.3. In vitro performance of the glucose-responsive insulin microspheres

**Figure 3.**
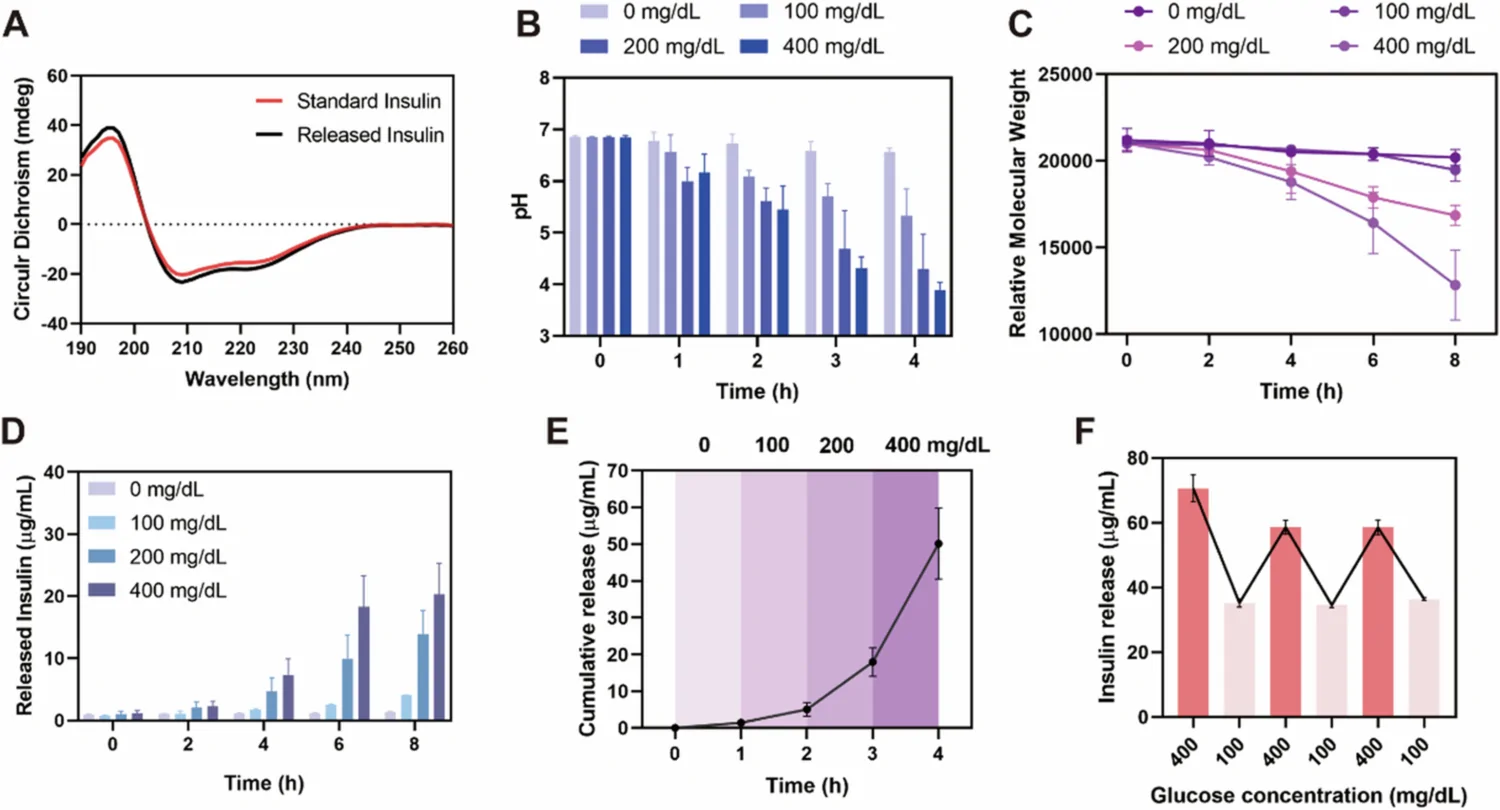
In vitro performance of the glucose-responsive insulin microspheres. (A) CD spectra of insulin released from microspheres compared with standard insulin. (B) Time-dependent pH changes of microspheres in PBS and glucose solutions (100, 200, and 400 mg/dL). Data are presented as mean ± s.d. (*n* = 3). (C) GPC analysis of the PLGA/Ac-dex matrix of microspheres following incubation in glucose solutions at concentrations of 0, 100, 200, and 400 mg/dL. Samples were collected at 0, 2, 4, 6, and 8 hours, and the weight-average molecular weight (Mw, Da) of the matrix is presented at each indicated time point. Data are presented as mean ± s.d. (*n* = 3). (D) Cumulative insulin release profiles from microspheres (Ac-dex: PLGA = 3:1) in response to different glucose concentrations (0, 100, 200, and 400 mg/dL). Data are presented as mean ± s.d. (*n* = 3). (E) Dynamic insulin release under stepwise glucose stimulation: microspheres were dispersed in PBS, and glucose concentrations were sequentially increased (0→100→200→400 mg/dL) at 1 hour intervals. Data are presented as mean ± s.d. (*n* = 3). (F) Pulsatile insulin release in response to alternating high (400 mg/dL) and low (100 mg/dL) glucose levels (1 hour per switch). Data are presented as mean ± s.d. (*n* = 3).

The bioactivity of encapsulated insulin was verified by circular dichroism (CD) spectroscopy. As shown in Figure 3A, the secondary structure curves of released insulin and standard insulin were nearly identical, with characteristic minima at 209 nm and 222 nm, corresponding to *α*-helical structures (Pocker and Biswas 1980; Du et al. 1982). These results confirm that the secondary structure and bioactivity of insulin remained unaltered after encapsulation. The enzymatic activity and leakage of the co‑encapsulated GOx and CAT were monitored over 14 days. As shown in Fig. S7A and S7B, both enzymes exhibited high residual activities during the first 5 days (GOx: 86.0 ± 6.2%; CAT: 76.8 ± 7.2%), with cumulative enzyme leakage below 5% over the same period (Fig. S7C). A gradual decline in enzymatic activity was observed thereafter, accompanied by increased enzyme leakage (10.1% ± 1.3% at day 14). These results confirmed that the GOx–catalase enzymatic cascade remained functionally intact during the initial 5‑day period, with over 85% activity retention and less than 5% enzyme leakage, which is considered sufficient to support the subsequent glucose‑triggered responses in vitro.

To validate the glucose-responsive controlled insulin release performance of the microspheres, we first measured the pH changes of the medium at different glucose concentrations (0, 100, 200, and 400 mg/dL). Here, blood glucose levels were categorized as follows: 100 mg/dL (normal), 200 mg/dL (hyperglycemic threshold), and 400 mg/dL (typical hyperglycemia), with PBS serving as the control. As demonstrated in Figure 3B, at time zero all solutions showed a pH value of 6.8. After 1 hour, the glucose concentration influenced the pH, as glucose was oxidized by GOx to gluconic acid. Consequently, the solution pH exhibited a concentration-dependent decrease; after 4 hours of incubation, the 400 mg/dL glucose group showed a 40.8% reduction compared with the PBS control. These results confirm the preserved enzymatic activity of GOx and establish the pH-response basis for the subsequent glucose-triggered release.

To further establish the mechanistic link between glucose-induced pH reduction and insulin release, we measured the weight-average molecular weight (Mw) of the polymer matrix within the microspheres by gel permeation chromatography (GPC) after incubation under varying glucose concentrations over 8 hours (Figure 3C). A time-dependent reduction in Mw was observed across all groups, confirming progressive polymer hydrolysis. Notably, higher glucose concentrations accelerated the Mw decline: after 8 hours, the Mw values were 20,190 ± 446, 19,476 ± 680, 16,860 ± 572, and 12,825 ± 1,976 Da at 0, 100, 200, and 400 mg/dL glucose, respectively. This glucose-accelerated degradation is attributable to the hydrolysis of acetal bonds within Ac-dex, producing acidic species that further promote autocatalytic PLGA chain scission. The consequent increase in matrix porosity would reduce diffusional barriers, and is therefore expected to facilitate insulin efflux. These degradation profiles thus provide mechanistic evidence that supports the glucose-responsive release behavior observed in the subsequent release experiments.

We further quantified insulin release under varying glucose conditions to directly assess the glucose-responsive behavior. Insulin release was measured at specific time points in gradient glucose solutions (0–400 mg/dL). As shown in Figure 3D, the cumulative insulin release after 8 hours demonstrated a pronounced glucose concentration-dependent profile, with measured values of 1.3 ± 0.2, 4.0 ± 0.1, 13.9 ± 3.8, and 20.3 ± 5.0 µg/mL at glucose concentrations of 0, 100, 200, and 400 mg/dL, respectively. Notably, the insulin release at 400 mg/dL glucose was 15.5-fold higher than that observed at 100 mg/dL, clearly indicating a dose-responsive release pattern. Although the release kinetics under high glucose (400 mg/dL) were not extremely rapid, with a lag phase prior to significant insulin efflux, this relatively moderate profile may help to minimize the risk of hypoglycemia—a critical safety concern in glucose-responsive insulin delivery systems. Despite the moderate release kinetics observed in vitro, the microspheres were able to reduce severely elevated blood glucose to the normoglycemic range within 1 hour, as will be further validated in the subsequent in vivo study. Once normoglycemia was achieved, the system sustained long-term glycemic stability. Furthermore, when subjected to sequential glucose concentration increments (0→100→200→400 mg/dL), the system displayed progressively enhanced insulin release kinetics, with corresponding release rates of 1.4 ± 0.6, 3.6 ± 2.4, 12.9 ± 2.1, and 32.2 ± 8.9 µg/mL·h, as quantitatively determined through slope analysis in Figure 3E. To simulate dynamic glycemic fluctuations in vivo, a pulsatile release experiment was conducted by alternating the microspheres between 400 and 100 mg/dL glucose solutions. The release profile (Figure 3F) displayed distinct pulsatile fluctuations, demonstrating the microspheres' ability to dynamically adjust insulin release in real-time according to ambient glucose levels.

The observed correlation between the glucose-accelerated matrix degradation (Figure 3C) and the enhanced insulin release (Figure 3D–F) suggests that polymer erosion actively contributes to the release kinetics. Higher glucose concentrations led to greater Mw reduction and correspondingly higher cumulative release, which is consistent with a mechanism wherein degradation increases matrix permeability and reduces diffusional resistance for insulin. The Mw continued to decline throughout the 8 h observation period without reaching a plateau, indicating that degradation was ongoing and may have continuously modulated the release profile. These findings support that matrix degradation is a contributing factor to the glucose-responsive release. Collectively, these in vitro experiments confirm the microspheres' capacity for glucose-responsive insulin delivery.

### 4.4. Therapeutic effect of glucose-responsive microspheres in STZ-induced T1D mice

**Figure 4.**
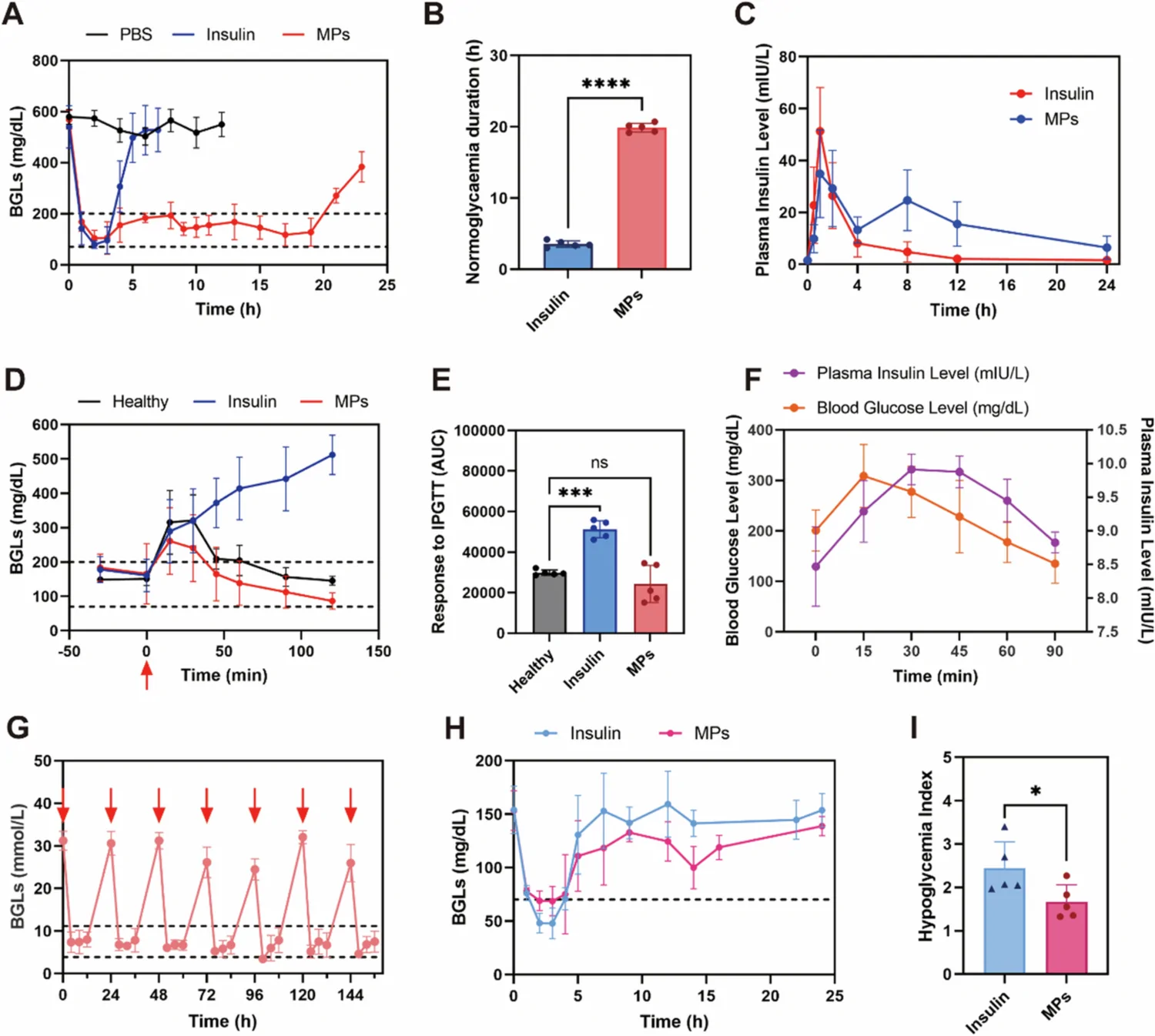
Therapeutic effect of glucose-responsive microspheres in STZ-induced T1D mice. (A) Blood glucose levels in diabetic mice treated with PBS, free insulin, or insulin-loaded microspheres. Data are presented as mean ± s.d. (*n* = 5). (B) Duration of normoglycemia (blood glucose ≤ 200 mg/dL) maintained by insulin and microsphere groups in (A). Data are presented as mean ± s.d. (*n* = 5). Two-tailed Student's t-test was used for statistical analysis. ****P < 0.0001. (C) Plasma insulin levels in T1D mice following a single subcutaneous injection of free insulin or insulin-loaded microspheres. Blood samples were collected at the indicated time points post-injection. Data are presented as mean ± s.d. (*n* = 5). (D) Intraperitoneal glucose tolerance tests (IPGTT) in diabetic mice treated with insulin or microspheres, with healthy mice as controls. Red arrow indicates glucose injection time (dose: 1.5 g/kg). Data are presented as mean ± s.d. (*n* = 5). (E) Area under the curve (AUC) analysis of glucose tolerance tests in (D). Data are presented as mean ± s.d. (*n* = 5). Two-tailed Student's t-test was used for statistical analysis. *P* > 0.05, not significant, ***p < 0.001. (F) Plasma insulin levels during IPGTT in diabetic mice. Glucose (3 g/kg) was injected at 0 hour (arrow). Data are presented as mean ± s.d. (*n* = 5). (G) Changes of blood glucose in T1D mice after subcutaneous injection of microspheres at 8: 00 every morning for one week. The red arrow indicates the administration time. Blood glucose was measured before administration every day. Data are presented as mean ± s.d. (*n* = 5). (H) Blood glucose profiles of healthy mice after treatment with insulin or microspheres (The injection doses of insulin and microspheres are the same as above). Data are presented as mean ± s.d. (*n* = 5). (I) Hypoglycemic index calculated for healthy mice in (H). The hypoglycemic index = [(initial glucose - nadir glucose)/time to nadir]. Data are presented as mean ± s.d. (*n* = 5). Two-tailed Student's t-test was used for statistical analysis. *P < 0.05.

The hypoglycemic effect of the microspheres was evaluated in STZ-induced diabetic C57BL/6J mice (male, 6–8 weeks, 20–23 g) (Su et al. 2021; Muddineti and Omri 2022). Diabetic mice were randomly divided into three groups receiving different subcutaneous injections while maintaining free access to food and water. As demonstrated in Figure 4A, PBS-treated control mice exhibited sustained hyperglycemia throughout the observation period. While free insulin administration (10 mg/kg) rapidly reduced blood glucose to normoglycemic levels (79.2 ± 15.0 mg/dL) within 2 hours, this effect was transient, with glucose levels returning to hyperglycemic range within 5 hours. In marked contrast, microsphere-treated mice (20 mg/kg) not only achieved effective glycemic reduction (minimum glucose level: 104.0 ± 30.8 mg/dL at 2 hours) but more importantly maintained prolonged normoglycemia for 20 hours post-administration. Quantitative comparison of therapeutic duration (Figure 4B) revealed that microspheres extended normoglycemic maintenance to 19.9 ± 0.6 hours – a 5.5-fold improvement over free insulin's 3.6 ± 0.5 hours duration, conclusively demonstrating the superior sustained-release performance of the microsphere formulation. Then, by simulating the three meals a day of humans, a fixed feeding time was given to diabetic mice to explore the therapeutic effect of the microspheres. The microspheres were subcutaneously injected once after the first meal, and insulin was subcutaneously injected after each meal. As shown in Fig. S8, it can be observed that the injection of insulin produced a transient blood glucose-lowering effect. After each meal, the blood glucose level of the diabetic mice increased again to the hyperglycemic level. Insulin could only be injected after each meal to treat diabetes, which led to significant fluctuations in the blood glucose of the mice within a day. In the group of mice injected with microspheres, the blood glucose level decreased after a single injection. After each subsequent meal, the blood glucose of the mice increased slightly but basically remained within the normal range. Compared with the mice treated with insulin, the blood glucose level of the mice in the microsphere group was more stable.

To evaluate the pharmacokinetic behavior of the microsphere formulation, plasma insulin concentrations were measured over time following subcutaneous administration of free insulin or insulin-loaded microspheres (Figure 4C). In the free insulin group, insulin levels rose sharply to a peak of 49.6 ± 18.9 mIU/L at 1 hour post-injection, followed by a rapid decline to baseline within 4 hours. In contrast, the microsphere group exhibited a distinctly different pharmacokinetic profile, with insulin levels reaching a moderate peak of 36.9 ± 18.3 mIU/L at 1–2 hours. Notably, after the initial decline, a secondary elevation in insulin levels was observed in several mice at 8 hours. This phenomenon may reflect the glucose-responsive nature of the microspheres, whereby the subsequent rise in blood glucose, occurring following the waning of the initial therapeutic effect, triggers additional insulin release. This suggests the capacity of the system to modulate insulin output in response to glycemic fluctuations. Thereafter, insulin levels remained relatively stable before gradually returning to baseline by 24 hours, consistent with the observed glycemic profiles. Overall, compared with free insulin, the microsphere formulation provides a prolonged and adaptive release profile, which may contribute to reduced hypoglycemic risk and more durable glycemic control.

To evaluate real-time glucose responsiveness, we conducted IPGTT (glucose dose: 1.5 g/kg) when treated mice achieved normoglycemia, with healthy mice serving as controls (Figure 4D). Healthy control mice exhibited transient hyperglycemia that rapidly normalized, as expected. Mice treated with free insulin displayed rapid hyperglycemic spikes following glucose challenge, ultimately reverting to their baseline hyperglycemic state. In contrast, microsphere-treated mice showed only modest glucose elevation (peak: 261.4 ± 97.0 mg/dL at 15 minutes post-challenge) followed by rapid normalization (165.2 ± 69.6 mg/dL by 60 minutes), maintaining stable normoglycemia thereafter. Corresponding AUC analysis (Figure 4E) quantitatively compared the glycemic control capacity between groups. Statistical analysis revealed that insulin-treated mice had 1.7-fold higher AUC than healthy controls, demonstrating impaired glucose counterregulation. Remarkably, microsphere-treated mice achieved glycemic control parameters comparable to healthy mice, indicating preserved physiological responsiveness to glucose challenges and superior maintenance of glucose homeostasis. During IPGTT, plasma insulin levels dynamically correspond to glucose fluctuations. To improve the detectability of changes in insulin response, we increased the glucose excitation concentration (glucose dose: 3 g/kg). Microsphere-treated mice exhibited glucose-dependent insulin release. As shown in Figure 4F, when blood glucose increased in mice after intraperitoneal injection of glucose, plasma insulin levels increased immediately thereafter, reaching 9.9 ± 0.2 mIU/L at 30 minutes. Subsequently, insulin acted to induce a decrease in blood glucose in mice, and the insulin levels decreased accordingly. Plasma insulin increased during hyperglycemia and decreased when glucose was normalized, demonstrating closed-loop function.

We then performed a one-week continuous subcutaneous injection of insulin microspheres to observe the continuous administration of microspheres. The blood glucose monitoring of T1D mice is shown in Figure 4G, which is in line with the results of the single administration described above. Normal blood glucose could be maintained in the mice after each day's administration, and the drug was not effective before the next day, and the injection of microspheres was continued to achieve the purpose of long-term stabilization of blood glucose.

To assess whether the microspheres release insulin under normoglycemic conditions and induce hypoglycemic symptoms, normoglycemic mice were administered free insulin or insulin-loaded microspheres at the same doses as those used in the therapeutic groups. Insulin-treated mice developed severe hypoglycemia with neuroglycopenic symptoms, including lethargy and seizures, while microsphere-treated mice, despite a modest reduction in blood glucose, remained within the normoglycemic range throughout the observation period and exhibited no typical hypoglycemic symptoms (Figure 4H). The hypoglycemic index was significantly lower for microspheres (Figure 4I), indicating a reduced hypoglycemic tendency.

### 4.5. In vivo biodistribution of Glucose-responsive microspheres

**Figure 5.**
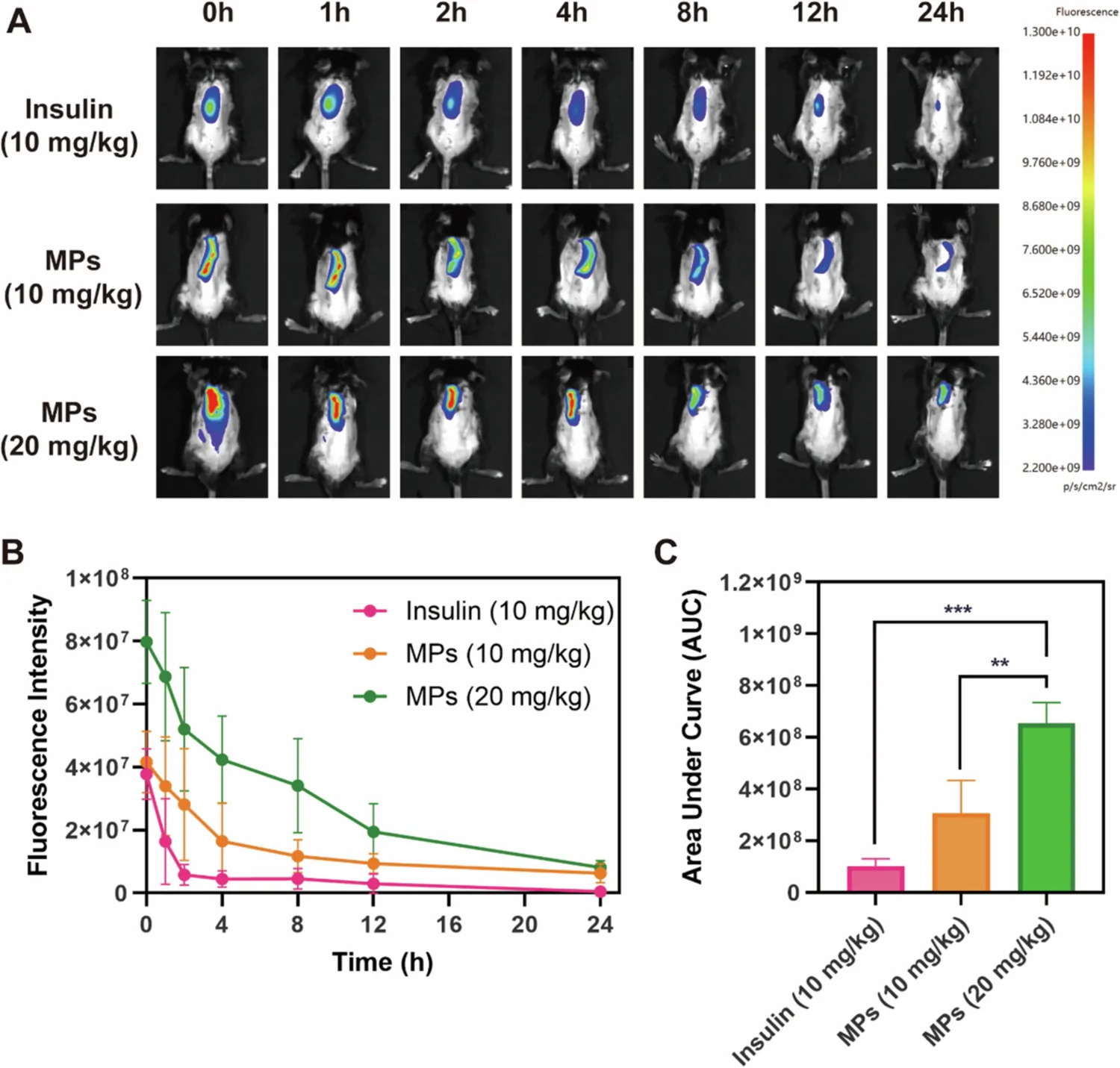
In vivo biodistribution of Glucose-responsive microspheres. (A) Fluorescence images of diabetic mice (*n* = 3) at 0, 1, 2, 4, 8, 12, and 24 hours post-injection of free insulin (10 mg/kg) or insulin-loaded microspheres (10, 20 mg/kg insulin-equivalent dose). Both microsphere-loaded and free insulin were labeled with Cy5 (Cy5-insulin). (B) Quantitative fluorescence intensity profiles corresponding to (A). Data are presented as mean ± s.d. (*n* = 3). (C) AUC of fluorescence change curve. Data are presented as mean ± s.d. (*n* = 3). One-way ANOVA with Tukey's post-hoc tests were used for statistical analysis. **P < 0.01, ***P < 0.001.

Fluorescence imaging of Cy5-labeled insulin following subcutaneous administration in diabetic mice (Figure 5A) demonstrated significantly prolonged retention of microsphere-encapsulated insulin compared to free insulin controls (10 mg/kg), with the therapeutic dose (20 mg/kg) exhibiting further enhanced persistence. Quantitative analysis revealed dose-dependent pharmacokinetic profiles, where fluorescence intensity curves (Figure 5B) confirmed sustained release characteristics, and corresponding AUC measurements (Figure 5C) showed 2.1-fold (10 mg/kg) and 6.4-fold (20 mg/kg) increases in systemic exposure relative to free insulin. These findings conclusively demonstrate the superior in vivo retention and prolonged therapeutic action of glucose-responsive insulin microspheres.

### 4.6. Biocompatibility evaluation of glucose-responsive microspheres

**Figure 6.**
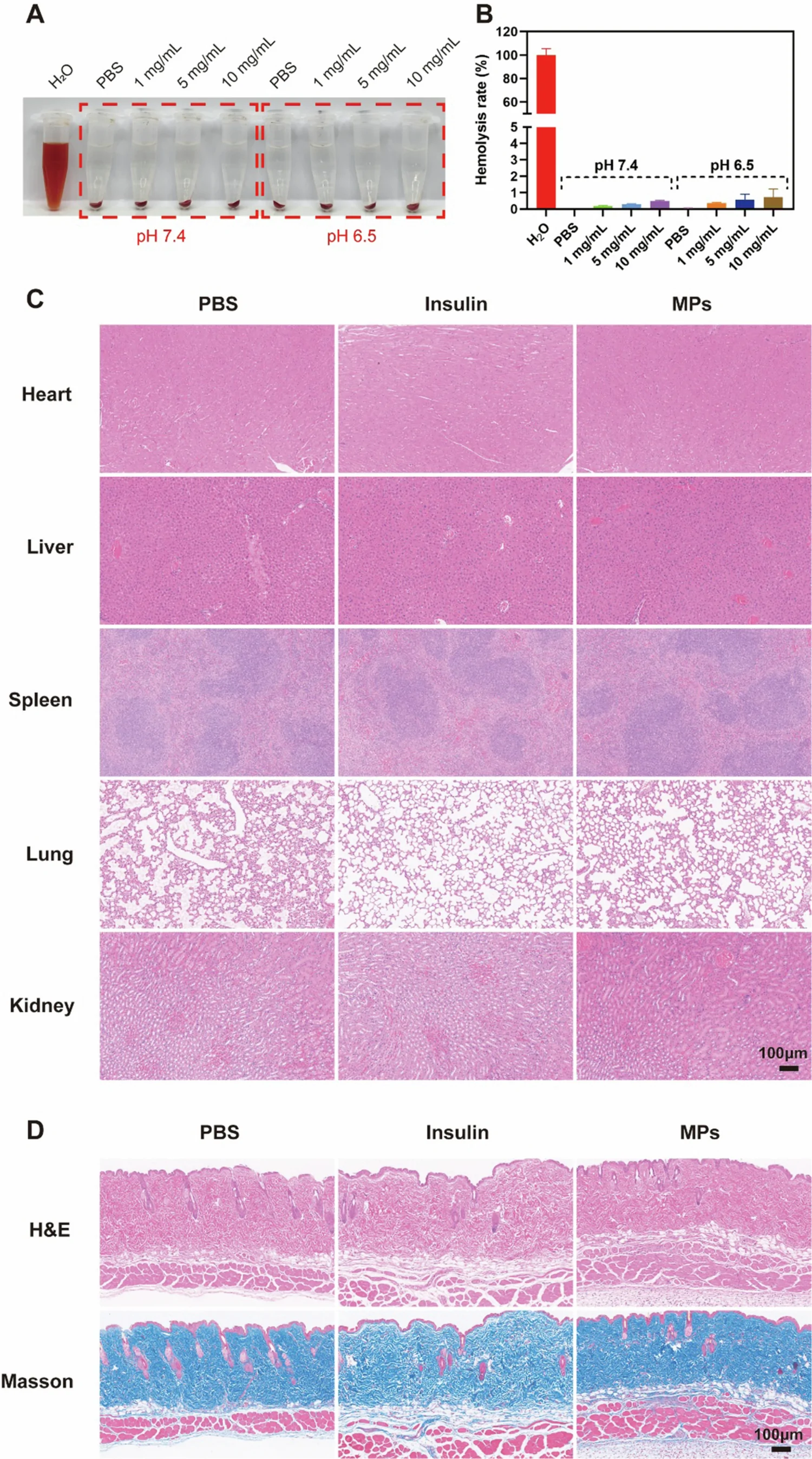
Biocompatibility evaluation of glucose-responsive microspheres. (A) Hemolysis assay: photographs of red blood cell suspensions after centrifugation following 2‑hour incubation with microspheres at increasing concentrations under pH 7.4 and pH 6.5, with PBS as negative control and distilled water as positive control. (B) Hemolysis rates of red blood cells after incubation with microspheres at varying concentrations under neutral (pH 7.4) and acidic (pH 6.5) conditions. (C) H&E staining of heart, liver, spleen, lung, and kidney tissues from diabetic mice (*n* = 3) after one‑month treatment with PBS, free insulin (10 mg/kg), or insulin-loaded microspheres (20 mg/kg insulin-equivalent dose, administered once daily). Scale bar: 100 µm. (D) H&E and Masson's trichrome staining of the injection site skin from the same treatment groups as in (C). Scale bar: 100 µm.

Hemolysis assays were performed to evaluate the hemocompatibility of the microspheres. Given that hyperglycemia may create a local acidic microenvironment, additional tests were conducted at pH 6.5, which represents the most extreme physiologically relevant condition the microspheres could encounter, as systemic blood pH is tightly maintained above 6.8 even under severe acidosis. As shown in Figure 6A, marked hemolysis was evident in the positive control group, whereas no obvious hemolysis was observed in any microsphere-treated groups, which were comparable to the negative control. Figure 6B shows that hemolysis rates increased in a concentration-dependent manner under both neutral and acidic conditions; although marginally higher at pH 6.5 than at pH 7.4, the rates remained below 2% for all groups, well within the 5% acceptability criterion of ISO 10993‑4. Collectively, these results confirm that the microspheres do not induce appreciable hemolysis even at pH 6.5, indicating that the potentially acidic microenvironment in diabetic tissues does not compromise their hemocompatibility.

Histological evaluation of major organs via H&E staining (Figure 6C) revealed normal tissue architecture across all groups after one-month treatment, with no obvious inflammatory infiltration observed in PBS-treated diabetic mice. Similarly, both insulin and microsphere groups exhibited preserved hepatic structure with only minimal inflammatory cells, comparable to PBS controls.

To minimize local tissue damage from daily injections, subcutaneous administration sites were rotated across different dorsal skin regions throughout the treatment period. Skin sections from injection sites were examined by H&E and Masson's trichrome staining (Figure 6D). H&E staining showed no obvious epidermal hyperplasia in any of the three groups, and Masson's trichrome staining revealed no significant collagen deposition or dermal fibrosis across all groups. The microsphere group exhibited marginally higher local tissue responses in terms of epidermal thickness and collagen deposition relative to the insulin and PBS groups, but these differences did not constitute overt hyperplasia or fibrosis. Microsphere aggregates were visible at the subcutaneous injection sites, yet the associated tissue reactions remained mild and comparable to those in the other groups. Inflammatory cell infiltration was minimal to absent in all groups. Collectively, these findings indicate favorable local biocompatibility of the microsphere formulation, with no significant skin pathology induced by either repeated daily injections or the presence of microsphere aggregates.

**Figure 7.**
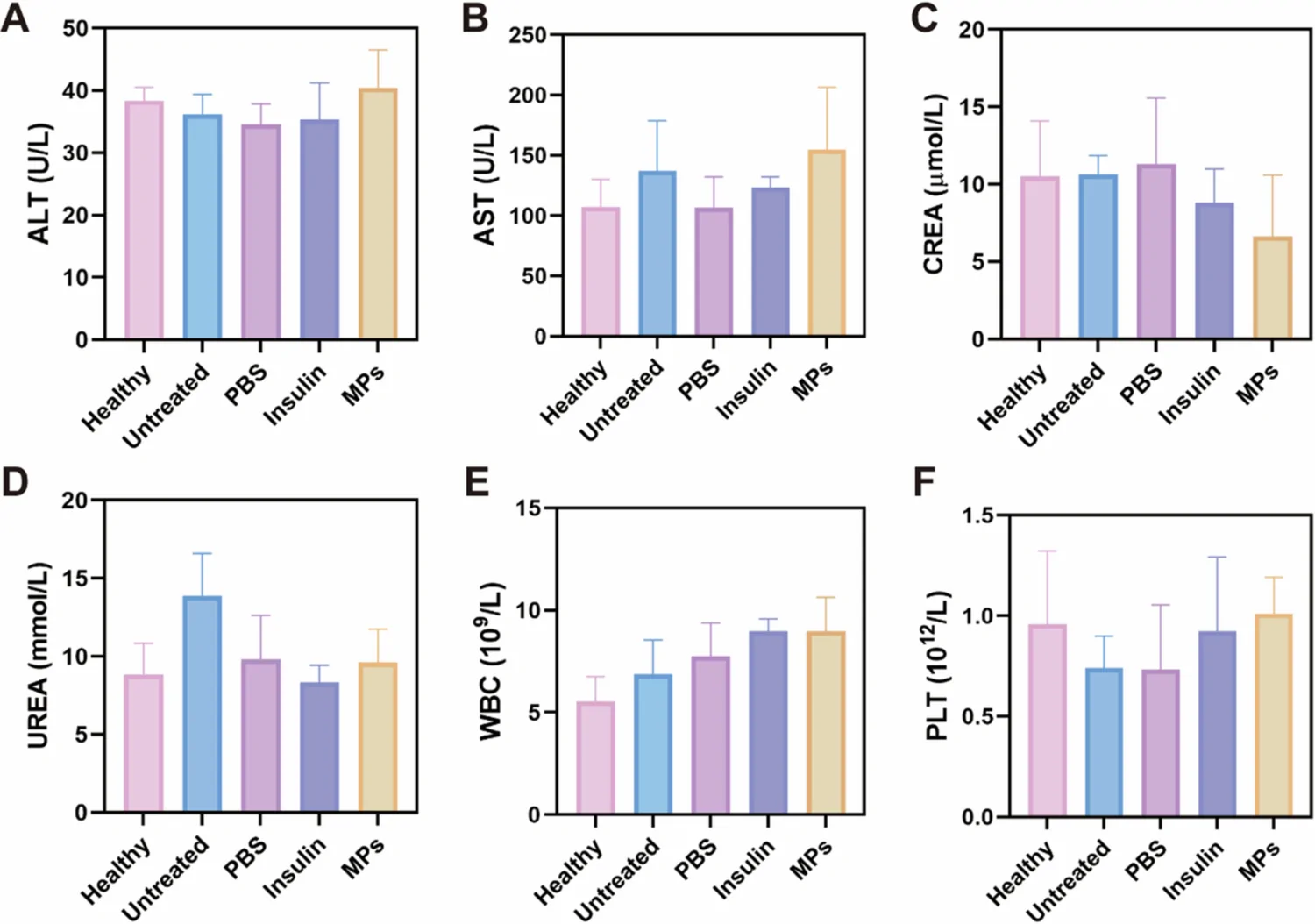
Hematological parameters of T1D mice after one month's treatment. Serum biochemical analysis of (A) ALT (alanine aminotransferase), (B) AST (aspartate aminotransferase), (C) CRE (creatinine), (D) UREA (urea), and blood cell count of (E) WBC (white blood cells), (F) PLT (platelets) in T1D mice. Healthy: Healthy mice, Untreated: Untreated T1D mice. PBS, Insulin (10 mg/kg), and MPs (20 mg/kg insulin-equivalent dose) were injected subcutaneously every day, and blood samples were taken after one month. Data are presented as mean ± s.d. (*n* = 5).

To further evaluate the long-term safety of the glucose-responsive microspheres, serum biochemical parameters (ALT, AST, CRE, UREA) and hematological indices (WBC, PLT) were measured in diabetic mice after one-month treatment, with healthy and untreated diabetic mice as controls. Compared with healthy controls, the microsphere-treated group showed slightly elevated ALT and AST levels (Figure 7A and B), yet these values were comparable to those of untreated diabetic and PBS groups, suggesting the increases reflected pre-existing STZ-induced hepatotoxicity rather than microsphere-related injury. CRE levels did not differ significantly across groups (Figure 7C). UREA levels were markedly elevated in untreated diabetic mice versus healthy controls (Figure 7D), confirming diabetes-induced renal impairment, whereas microsphere treatment normalized UREA to control levels. Notably, after one month of STZ induction, diabetic mice may have partially adapted to the altered physiological state, which could account for the lack of marked differences in several parameters relative to healthy controls. Hematological analysis showed WBC and PLT counts in the microsphere group within normal ranges and comparable to healthy controls (Figure 7E and F), with no signs of inflammation or coagulation abnormalities. Collectively, these results indicate that one-month repeated microsphere administration does not induce additional hepatic, renal, or hematological toxicity beyond the pre-existing diabetic pathology.

## 5. Conclusion

In this study, we developed a glucose-responsive insulin-loaded microsphere system using microfluidic technology, which integrates PLGA as a biocompatible matrix and Ac-dex as a pH-sensitive carrier, coupled with GOx for dynamic insulin release. The combination of these materials optimizes repetitive glucose-responsive performance, enabling the microspheres to function as a subcutaneous insulin depot for on-demand glycemic control. In vitro release assays demonstrated excellent glucose-dependent insulin release kinetics, while in vivo studies in STZ-induced diabetic mice confirmed sustained normoglycemia for over 20 hours. Notably, the microfluidic fabrication platform ensured high uniformity, stability, and batch-to-batch reproducibility of the microspheres under cost-effective conditions. This work not only provides a novel strategy for closed-loop insulin delivery but also extends the potential of pH-responsive microspheres to other disease models. Although our work provided a potential long term antidiabetic effect in STZ-induced diabetic mice, we recommend further investigation on an alternative biodegradable polymer-based design with faster degradation profile while maintaining biocompatibility.

## Supplementary Material

Supplementary Material Supplementary information.docx

## Data Availability

The authors confirm that the data supporting the findings of this study are available within the article and its supplementary materials.
