# Supplementary material for: Microfluidic-engineered glucose-responsive microparticle depot for smart insulin delivery
Source: Drug Deliv. 2026 Sep 3;33(1):2726633. doi: 10.1080/10717544.2026.2726633 (PMC13543840; doi:10.1080/10717544.2026.2726633)
Supplement: Supplementary file 1 [file IDRD_A_2726633_SM7658.docx]

**Supplementary information**

**Microfluidic-Engineered Glucose-Responsive Microparticle Depot for Smart Insulin Delivery**

Zhihan Li ^1^, YanWen Zhang ^1^ and Chuanpin Chen ^1, *^

^1^Department of Pharmacy, Xiangya School of Pharmaceutical Sciences, Central South University, Changsha, Hunan 410013, China; zhihanli0608@163.com; 224176@csu.edu.cn; [ccpin2000@hotmail.com](mailto:ccpin2000@hotmail.com)

*Corresponding author:

Chuanpin Chen.

Xiangya School of Pharmaceutical Sciences,

Central South University,

People's Republic of China.

ccpin2000@hotmail.com;

Tel.: +86-189-4204-6737

**Figure S1. Schematic of Ac-dex synthesis.**

**Figure S2. 1H-NMR analysis of dextran and Ac-dex.**

**Figure S3. FT-IR spectrum of dextran and Ac-dex.**

**Figure S4.** **The LC and EE of Microspheres of Ac-dex:PLGA ratios of 1:1, 3:1, 5:1, and 7:1.**

**Figure S5.** **Insulin release from the microspheres with various Ac-dex/PLGA weight ratios (1:1, 3:1, 5:1, and 7:1) was evaluated in glucose solutions with gradient concentrations.**

**Figure S6. In vitro release profiles of Ac-dex/PLGA microspheres with mass ratios of 3:1 and 5:1 in PBS (pH 7.4) at 37℃ over 14 days.**

**Figure S7. In vitro stability and leakage profiles of co-encapsulated enzymes over 14 days.**

**Figure S8. Blood glucose profiles of diabetic mice in response to a daily three-meal feeding regimen.**

Figure S1. Schematic of Ac-dex synthesis.

Figure S2. (A) ¹H-NMR spectrum of dextran in D₂O, 500 MHz. (B) ¹H-NMR spectrum of acetylated dextran (Ac-dex) in d-DMSO, 500 MHz.

Figure S3. (A) FT-IR spectrum of dextran. (B) FT-IR spectrum of Ac-dex.

Figure S4. The LC and EE of Microspheres of Ac-dex:PLGA ratios of 1:1, 3:1, 5:1, and 7:1. Data are presented as mean ± s.d. (n=3).

Figure S5. Insulin release from the microspheres with various Ac-dex/PLGA weight ratios (1:1, 3:1, 5:1, and 7:1) was evaluated in glucose solutions with gradient concentrations. Data are presented as mean ± s.d. (n=3).

Figure S6. In vitro release profiles of Ac-dex/PLGA microspheres with mass ratios of 3:1 and 5:1 in PBS (pH 7.4) at 37℃ over 14 days. Data are presented as mean ± s.d. (n=3).

Figure S7. In vitro stability and leakage profiles of co-encapsulated enzymes over 14 days. (A) Relative activity of GOx in the microspheres. Data are presented as mean ± s.d. (n=3). (B) Relative activity of CAT in the microspheres. Data are presented as mean ± s.d. (n=3). (C) Cumulative leakage of total protein (enzymes) from the microspheres into the release medium. All measurements were performed in PBS (pH 7.4) at 37 °C. Data are presented as mean ± s.d. (n=3).

Figure S8. Blood glucose profiles of diabetic mice in response to a daily three-meal feeding regimen. Ac-dex/PLGA microspheres (MPs, dose: 20 mg/kg) were subcutaneously administered after the first meal (blue arrows), followed by bolus insulin injections (dose: 10 mg/kg each) after each meal (black arrows). The red shaded areas indicate the feeding periods.
